# Effect of at-home and in-office bleaching on microleakage of class V composite resin restorations using different types of universal adhesives: An in vitro study

**DOI:** 10.34172/joddd.2023.40820

**Published:** 2023-12-30

**Authors:** Mehdi Abed Kahnamouei, Soodabeh Kimyai, Katayoun Katebi, Mohammad Esmaeel Ebrahimi Chaharom, Mehdi Daneshpooy, Mahmoud Bahari, Mahshid Moradi

**Affiliations:** ^1^Department of Esthetic and Restorative Dentistry, Faculty of Dentistry, Tabriz University of Medical Sciences, Tabriz, Iran; ^2^Department of Oral and Maxillofacial Medicine, Faculty of Dentistry, Tabriz University of Medical Sciences, Tabriz, Iran; ^3^Dental and Periodontal Research Center, Tabriz University of Medical Sciences, Tabriz, Iran

**Keywords:** Composite resins, Dental bonding, Dental leakage, Tooth bleaching

## Abstract

**Background.:**

When bleaching agents contact dental structures, they act on restorative materials and adhesive interfaces. This study investigated the effect of "at-home" and "in-office" bleaching on the microleakage of composite resin restorations performed with different universal adhesives in self-etch and etch-and-rinse modes.

**Methods.:**

Class V cavities were prepared in 132 premolars. The samples were divided into four groups (n=33). All Bond Universal adhesive was used in the first and second groups, and G-Premio Bond adhesive was used in the third and fourth groups. The total-etch mode was used in the first and third groups, and the self-etch mode was used in the second and fourth groups. The samples were divided into three subgroups (n=11). In the first subgroup, home bleaching was used, and in the second subgroup, office bleaching was used. In the third subgroup, bleaching was not performed. The specimens were examined under a stereomicroscope for microleakage. Ordinal regression analysis was applied (*P*<0.05).

**Results.:**

The adhesive type, application method, and margin type significantly affected microleakage (*P*<0.05). The amount of microleakage in All Bond Universal adhesive was significantly higher than in G-Premio Bond adhesive. The chance of microleakage in the self-etch mode was almost twice as high as in the etch-and-rinse mode. The bleaching method did not significantly affect microleakage (*P*>0.05).

**Conclusion.:**

Based on the results of the microleakage test, bleaching after composite resin restorations did not significantly affect the microleakage of Class V restorations.

## Introduction

 Tooth color is one of the critical parameters in achieving the beauty of teeth, which can be improved with different methods and approaches, one of which is bleaching. Bleaching is an effective and conservative process performed using a chemical agent to oxidize organic pigments on the teeth.^1^ The two main protocols for bleaching vital teeth include home bleaching and office bleaching.^2^

 Since the bleaching material is placed in close contact with the tooth and any related restoration, it may adversely affect the natural structure of the tooth, bonding interface, and restoration material.^3^ Some studies have shown that bleaching gels negatively affect the bond strength of restored teeth. Weak marginal seal and low bond strength lead to microleakage, which causes bacteria, liquids, and ions to penetrate the tooth‒restoration interface.^4^ Microleakage is an important measure of success in all restorations. It is an indicator of pulp irritation and necrosis, sensitivity after restoration, and caries recurrence. The choice of adhesive type may affect the adhesion of restorative materials to dental tissues.^5^ Some studies have shown that one-step self-etch adhesives cause a greater increase in microleakage in the enamel margin after bleaching than two-step self-etch adhesives.^6^ However, some other studies that used a universal adhesive in both self-etch and total-etch methods concluded that etching and bleaching with 40% hydrogen peroxide does not affect the microleakage of enamel and dentin margins.^7^ Therefore, considering the contradictions in the past studies and the lack of sufficient information regarding the effect of bleaching on the microleakage of universal adhesives, the present study investigated the effects of home bleaching and office bleaching on the microleakage of class five composite resin restorations using two universal adhesive systems.

## Methods

 In this in vitro study, two cavities (264 cavities in total) were prepared in each buccal and lingual aspect of 132 human premolar teeth. Inclusion criteria included sound teeth without caries from patients aged 25 to 35 years that were extracted for orthodontic reasons. Teeth with caries, cracks, and teeth extracted for a long time were excluded. According to the results of Klein et al,^8^ the mean ± standard deviation of the microleakage score in the Single Bond adhesive group was 0.74 ± 1.86, and in the Clearfil SE bond adhesive group, it was 0.053 ± 2.1. Considering the first type error at 0.05 and 85% power, 110 cavities in each adhesive group were obtained. To increase the study’s validity, 20% was added to the sample size, which was finally considered 132 cavities in each type of adhesive. Since each type of adhesive was used in both self-etch and total-etch methods, there were 66 cavities in each group. According to the bleaching method, which was performed in three methods, there were 22 cavities in each subgroup in the end. On the buccal and lingual surfaces of the teeth, Class V cavities with dimensions of approximately 2 mm occlusogingivally, 2 mm deep, and 4 mm mesiodistal wide were prepared with a 008 diamond fissure bur (Komet, Dental Burs, Rock Hill, USA) under water spray cooling. The burs were replaced after preparing 10 cavities.^6^ The cavities were prepared so that the enamel margin was 1 mm above the CEJ and the dentin margin was 1 mm below the CEJ. The margins of the cavities were not beveled, and the cavosurface angle was approximately 90 degrees. The samples were randomly divided into four groups of 33 (based on the type of adhesive system and the application method). In the first and second groups, All Bond Universal adhesive system (Bisco, Schaumburg, IL, USA) was used, and in the third and fourth groups, G-Premio Bond (GC, Tokyo, Japan) was used according to the manufacturers’ instructions. The classification and compositions of adhesives tested in the study are listed in Table 1.

**Table 1:** Compositions of universal adhesives tested in the study

**Material**	**Classification**	**Composition **
All-Bond Universal	Ultra-Mild (pH = 3.1)	10-MDP, 2-HEMA, BisGMA, Ethanol, Water, Photoinitiator
G-Premio Bond	Moderate (pH = 1.5)	10-MDP, 4-MET, MTDP, methacrylic acid ester, Silica, acetone, water, photoinitiators

10-MDP:10-methacryloyloxydecyl dihydrogen phosphate, 2-HEMA: 2-hydroxyethyl methacrylate; BisGMA: Bisphenol-A glycidyl dimethacrylate, 4-META: 4-methacryloxyethyl trimellitic acid, MDTP: Methacryloyloxydecyl dihydrogen thiophosphate.

 The cavities were prepared by the total-etch method in the first and third groups. The cavity walls were etched using 37% phosphoric acid (Ivocar, Vivadent, Liechtenstein) for 15 seconds. Then, the surface of the samples was washed for 15 s, and excess water was removed so that the surface of the dentin remained moist. In the second and fourth groups, the cavities were prepared by the self-etch method. The adhesive was used in each group according to the manufacturer’s instructions for 10 seconds, the solvent was vaporized by air pressure for 5 seconds, and the samples were cured for 20 seconds.

 All the cavities were restored with Spectrum shade A1 composite resin (Dentsply, Surrey, UK) by incremental technique, and each layer was cured for 20 seconds. Finally, finishing and polishing were performed with polishing discs (Soflex, 3M ESPE). After this stage, the samples were subjected to 500 cycles of thermocycling, with a temperature of 5 ± 2°C and 55 ± 2°C consecutively. Each thermal cycle lasted 80 s, which included placing the samples for 30 seconds at 5°C, 30 seconds at 55°C, and 20 seconds at room temperature.^9^ After this stage, the samples in each group were divided into three subgroups of 11: A (bleaching at home), B (bleaching in the office), and C (control).

 In subgroup A, the home bleaching gel 15% carbamide peroxide (Ultradent, South Jordan, Utah, USA) was used in contact with the teeth for 6 s daily for 10 days. After each use, the gel was removed with air spray, and the teeth were kept in an environment with 50% relative humidity for 10 days. In subgroup B, 40% hydrogen peroxide bleaching gel (Ultradent, South Jordan, Utah, USA) was used in the office, which remained in contact with the teeth for 15 min and then washed. On another day in the same week, the bleaching gel was used for the second time for 15 minutes (30 minutes in total).

 Subgroup C was not subjected to bleaching as a control subgroup. After this stage, the apical foramen was filled with self-curing glass ionomer and inlay wax, with little dimensional changes. All the tooth surfaces, including the apical foramen, except for the restoration surfaces and 1 mm around the restoration margins, were covered with two layers of varnish half an hour apart.

 Then, the samples were placed in a 2% methylene blue solution for 24 h at 23 °C.^6^ After washing and drying, they were cut by a diamond disc (Komet, 942200, GmbH, Lemgo, Germany) in the buccolingual direction from the middle of the restorations, in the direction of the longitudinal axis of the tooth and examined under a stereomicroscope at × 40 magnification.

**Figure 1:**
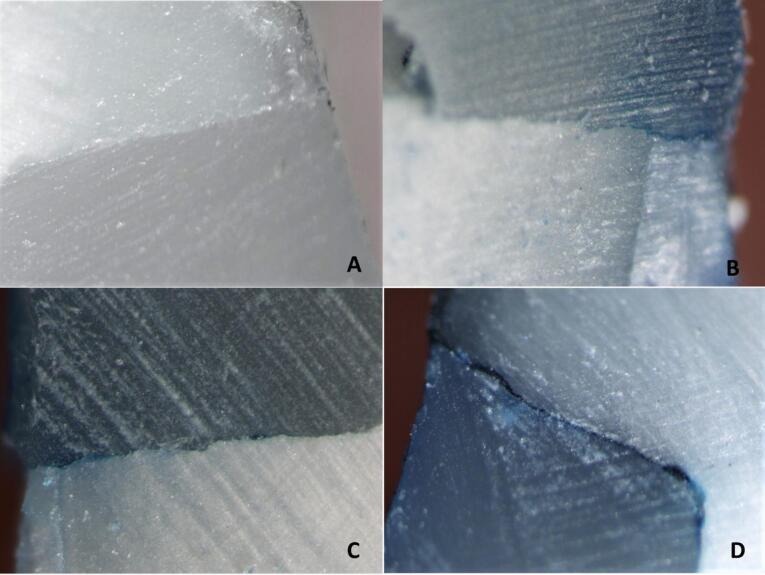
Different microleakage scores. A) Score 0. B) Score 1. C) Score 2. D) Score 3

 Grading of microleakage based on color penetration in the margins was as follows (Figure 1):

 0 = no dye penetration

 1 = penetration rate less than half the distance to the axial wall

 2 = penetration more than half the distance to the axial wall

 3 = penetration up to the axial wall and more.^8^

###  Statistical analysis 

 The results of the descriptive analysis were reported as frequency (percentage). The chi-square test was used to investigate two-by-two relationships, and ordinal regression analysis was used to investigate the effect of the type of intervention (home and office), type of adhesive, margin type, and conditioning techniques (self-etch and total-etch). All the analyses were performed using SPSS 26. The level of statistical significance was set at *P* < 0.05.

## Results

 The results indicated that the fitted regression model was statistically significant (χ^2^ = 51.753, df = 5, *P* < 0.001).

 Based on the results obtained from ordinal regression, the variables of adhesive type (*P* = 0.005), adhesive application mode (self-etch and total-etch) (*P* < 0.001), and margin type (*P* < 0.001) significantly affected microleakage (Table 2). By keeping the effect of other variables constant, All Bond Universal adhesive had a higher probability of microleakage compared to G-Premio Bond adhesive; to be more precise, the chance of being at a high level of microleakage for All Bond Universal adhesive compared to G-Premio Bond was almost 60% more (OR = 1.603, *P* = 0.005). The chance of being in a high level of microleakage in the self-etch mode was almost twice that of the total-etch mode (OR = 2.053, *P* ≤ 0.001). The type of margin, whether the dentin or enamel, significantly affected the amount of microleakage, so the dentinal margin had almost twice the chance of microleakage compared to the enamel margin (OR = 2.275, *P* ≤ 0.001). The intervention variable, or bleaching, keeping the effect of other factors constant, had no significant effect on the amount of microleakage (*P* = 0.171). The frequency (percentage) of microleakage score based on bleaching method, type of adhesive, two modes of self-etching and total etching, and margin type is presented in Table 3.

**Table 2:** The results of ordinal regression analysis to investigate the effect of each group on the amount of microleakage

**Variables**	**OR**	**SE**	***P*** ** value**	**95% confidence interval for OR**	
				**Lower bound**	**Upper bound**
Bleaching method					
At-home	1.323	0.204	0.171	0.886	1.974
In-office	1.336	0.204	0.156	0.895	1.994
Control	1	-	-	-	-
Adhesive					
All Bond Universal	1.603	0.168	0.005	1.154	2.225
G-Premio Bond	1	-	-	-	-
Adhesive application					
Self-etch	2.053	0.169	< 0.001	1.474	2.860
Total-etch	1	-	-	-	-
Margin					
Dentin	2.275	0.170	< 0.001	1.631	3.174
Enamel	1	-	-	-	-

OR: odds ratio, SE: standard error.

**Table 3:** Frequency (percentage) of microleakage score based on bleaching method, type of adhesive, two modes of self-etching and total etching, and margin type

		**Microleakage score**				***P*** ** value**
		**0**	**1**	**2**	**3**	
Bleaching method	At-home	58 (33.0%)	80 (45.5%)	27 (15.3%)	11 (6.3%)	0.168
	In-office	50 (28.4%)	95 (54.0%)	24 (13.6%)	7 (4.0%)	
	Control	63 (35.8%)	86 (48.9%)	25 (14.2%)	2 (1.1%)	
Adhesive	All Bond Universal	73 (27.7%)	133 (50.4%)	45 (17.0%)	13 (4.9%)	0.043
	G-Premio Bond	98 (37.1%)	128 (48.5%)	31 (11.7%)	7 (2.7%)	
Adhesive application	Self-etch	63 (23.9%)	144 (54.5%)	41 (15.5%)	16 (6.1%)	< 0.001
	Total-etch	108 (40.9%)	117 (44.3%)	35 (13.3%)	4 (1.5%)	
Margin	Dentin	69 (26.1%)	124 (47.0%)	57 (21.6%)	14 (5.3%)	< 0.001
	Enamel	102 (38.6%)	137 (51.9%)	19 (7.2%)	6 (2.3%)	

*P* value based on chi-square test.

## Discussion

 In recent years, bleaching has become a common treatment modality to remove surface stains and restore the beauty of teeth. Many bleaching materials, including hydrogen peroxide and carbamide peroxide, are used for bleaching.^10^ The mechanism of bleaching is based on a complex oxidation reaction in which oxygen free radicals penetrate the enamel and dentin due to their low molecular weight. The bleaching agent reacts with the pigments.^11^ This reaction opens the carbon rings of the pigments, turns these rings into more transparent intermediate chains, and results in tooth whitening.^12^ This study was designed and implemented to investigate the microleakage of class V composite resin restorations performed using two different types of universal adhesive in two modes: self-etch and etch-and-rinse after at-home and in-office bleaching.

 In the present study, thermocycling was used to simulate thermal changes in the oral cavity to evaluate the effect of bleaching on dental restorations in the mouth and artificial aging of the samples. Ten cycles of thermocycling on a sample is equivalent to oral conditions of one day.^13^ Therefore, 500 cycles of thermocycling performed in this study is equivalent to placing the samples in the patient’s mouth for 50 days.

 In the present study, two types of bleaching gel were applied to Class V composite resin restorations. The results showed that although the use of bleaching had a negative effect on microleakage, this effect was not significant, and no significant difference was found with the control group. Similarly, Iovan et al^7^ used G-Premio Bond universal adhesive in two self-etch and total-etch modes, concluding that the etching and bleaching strategy with 40% hydrogen peroxide did not affect marginal microleakage. Although the bleaching process might have affected the interface between the composite and the dental tissue, this effect was so small that the electron microscope did not detect it. Klein et al^8^ investigated the impact of two types of bleaching gel (15% Opalescence PF and 40% Opalescence Boost) on the microleakage of class V composite resin restorations with Adper Single Bond and Clearfil SE Bond adhesives. The results showed no significant difference in the microleakage between the two adhesive groups, and no significant difference was found between the two types of bleaching gel. However, in contrast to the present study, the teeth that were not bleached had a significantly lower microleakage score. Silva et al^14^ studied the effect of home and office bleaching on microleakage of Class V composite resin restorations prepared with Adper Single Bond adhesive. The results showed no significant difference between the control and bleached groups. However, in other studies, different findings have been reported. Bektas et al^15^ compared the microleakage of class V composite resin restorations with Clearfil SE Bond and Prime & Bond adhesives after applying 10% Opalescence PF and 38% Opalescence Boost bleaching gels. According to the results, the microleakage of composite resin restorations was different according to the bleaching methods used. However, no significant difference was observed in microleakage between the two adhesives in self-etch and total-etch modes. One of the main reasons for the difference in the results between Bektas and colleagues’ study and the present study is the difference in the percentage of bleaching gels and the method of implementation. In the Bektas and colleagues’ study, 10% Opalescence PF and 38% Opalescence Boost bleaching gels were used. In the in-office bleaching group, each restoration was exposed to Opalescence Boost for 15 minutes each time for 4 consecutive days, i.e., 60 minutes in total, and in the at-home bleaching group, each restoration was exposed for 8 hours a day for 10 days. In our study, the bleaching time with Opalescence Boost on each restoration was 30 minutes, and in the at-home bleaching group, it was 6 hours a day. Therefore, bleaching time affected the amount of microleakage.

 The results of the present study showed that the difference in microleakage between adhesive systems was significant. All Bond Universal adhesive had a higher probability of microleakage compared to G-Premio Bond adhesive. Due to the small number of studies on the effect of adhesives on the microleakage of composite restorations after bleaching and the lack of exact similarity of any of the studies with the current research, it is not possible to make a proper comparison between this study and other studies about these two types of adhesives.

 MDP is one of the functional monomers that allow using a universal adhesive with various etching techniques.^16^ The 10-methacryloyloxydecyl dihydrogen phosphate (10-MDP) monomer has a high potential to interact with hydroxyapatite. The bond created with adhesives containing 10-MDP appears to be very stable. The ability to etch is related to the pH of the applied substrate (the pH of G-Premio Bond adhesive is lower; as a result, it has more demineralization power), the monomer composition, and the bonding potential of functional monomers. 4-methacryloxyethyl trimellitic acid (4-META) works more effectively than HEMA to increase the bond strength of enamel and dentin. Although 2-hydroxyethyl methacrylate (HEMA) improves bond strength, the adhesives without HEMA are preferred because of their hydrophilicity.^17^ It was concluded in the present study that the probability of microleakage in the self-etch mode was almost twice that of the total-etch mode. As an explanation, the durability of the bond between the composite resin restorations and tooth tissues requires sufficient demineralization and optimal penetration of the bonding agent to enamel and dentin. These factors may be crucial for self-etch adhesives.^6^

 In a study by Iovan et al,^7^ the universal adhesive G-Premio Bond was used in two modes: self-etch and total-etch. Although the results showed a greater tendency to microleakage in the self-etch group, this difference was not significant, and the microleakage of the composite resin restorations after bleaching did not reveal a significant difference between the enamel and cervical margins of the etched samples. Therefore, the etching process before applying the universal bond did not increase the marginal seal of composite resin restorations after bleaching.

 Also, the results of the present study showed that microleakage in the dentin margin was significantly higher than in the enamel margin, which can be interpreted by the difference in the composition of the tooth in the cervical and enamel margins. In fact, the cement near the CEJ is more organic and thinner. Therefore, it is easily affected by the chemicals in bleaching agents. Hajilou et al^18^ investigated the effect of 40% hydrogen peroxide bleaching on the microleakage of composite resin restorations, reporting that bleaching after restoration increases the microleakage, with significantly greater microleakage in gingival margins.

## Limitations

 Clinical conditions and contamination with saliva, bacteria, and their protein synthesis might affect test results since the presence of natural saliva causes remineralization after bleaching and may affect microleakage. Therefore, the results might not be generalizable to clinical conditions.

 Extensive clinical research with different conditions is needed to determine the microleakage of composite resin restorations after tooth bleaching.

## Conclusion

 The results showed that the type of adhesive system significantly affected microleakage. Microleakage with All Bond Universal adhesive was significantly higher than with G-Premio Bond adhesive. The type of margin also had a significant effect on microleakage; the enamel margin had less microleakage than the cervical margin. Despite this, the method of bleaching did not have a significant impact on microleakage.

## Acknowledgments

 The Authors thank the staff of the Dental and Periodontal Research Center for their cooperation in this research.

## Competing Interests

 The authors declare no competing interests.

## Ethical Approval

 The Ethics Committee of Tabriz University of Medical Sciences approved this study under the code IR.TBZMED.REC.1400.1209.

## Funding

 Dental and Periodontal Research Center, Tabriz University of Medical Sciences, Tabriz, Iran.
